# Associations between unilateral amblyopia in childhood and cardiometabolic disorders in adult life: a cross-sectional and longitudinal analysis of the UK Biobank

**DOI:** 10.1016/j.eclinm.2024.102493

**Published:** 2024-03-07

**Authors:** Siegfried Karl Wagner, Vasiliki Bountziouka, Pirro Hysi, Jugnoo Sangeeta Rahi, UK Biobank Eye & Vision Consortium, Naomi Allen, Tariq Aslam, Denize Atan, Konstantinos Balaskas, Sarah Barman, Jenny Barrett, Paul Bishop, Graeme Black, Tasanee Braithwaite, Roxana Carare, Usha Chakravarthy, Michelle Chan, Sharon Chua, Alexander Day, Parul Desai, Bal Dhillon, Andrew Dick, Alexander Doney, Cathy Egan, Sarah Ennis, Paul Foster, Marcus Fruttiger, John Gallacher, David (Ted) Garway-heath, Jane Gibson, Jeremy Guggenheim, Chris Hammond, Alison Hardcastle, Simon Harding, Ruth Hogg, Pirro Hysi, Pearse Keane, Sir Peng Tee Khaw, Anthony Khawaja, Gerassimos Lascaratos, Thomas Littlejohns, Andrew Lotery, Robert Luben, Phil Luthert, Tom Macgillivray, Sarah Mackie, Savita Madhusudhan, Bernadette Mcguinness, Gareth Mckay, Martin Mckibbin, Tony Moore, James Morgan, Eoin O'sullivan, Richard Oram, Chris Owen, Praveen Patel, Euan Paterson, Tunde Peto, Axel Petzold, Nikolas Pontikos, Jugnoo Rahi, Alicja Rudnicka, Naveed Sattar, Jay Self, Panagiotis Sergouniotis, Sobha Sivaprasad, David Steel, Irene Stratton, Nicholas Strouthidis, Cathie Sudlow, Zihan Sun, Robyn Tapp, Dhanes Thomas, Emanuele Trucco, Adnan Tufail, Ananth Viswanathan, Veronique Vitart, Mike Weedon, Katie Williams, Cathy Williams, Jayne Woodside, Max Yates, Yalin Zheng, Mervyn Thomas

**Affiliations:** a Institute of Ophthalmology, University College London, London, UK; b NIHR Biomedical Research Centre at Moorfields Eye Hospital and UCL Institute of Ophthalmology London, UK; c Computer Simulation, Genomics and Data Analysis Laboratory, Department of Food Science and Nutrition, University of the Aegean, Greece; d Great Ormond Street Institute of Child Health, University College London, London, UK; e Cardiovascular Research Centre, Department of Cardiovascular Science, University of Leicester, Leicester, UK; f Section of Ophthalmology, School of Life Course Sciences, King's College London, London, UK; g Department of Twin Research and Genetic Epidemiology, King's College London, London, UK; h Great Ormond Street Hospital NHS Foundation Trust, London, UK; i Ulverscroft Vision Research Group, University College London, London, UK; j NIHR Biomedical Research Centre at UCL Great Ormond Street Institute of Child Health and Great Ormond Street Hospital, London, UK

**Keywords:** Amblyopia, Neurodevelopment, Cardiometabolic dysfunction, Retina, Oculomics

## Abstract

**Background:**

Amblyopia is a common neurodevelopmental condition and leading cause of childhood visual impairment. Given the known association between neurodevelopmental impairment and cardiometabolic dysfunction in later life, we investigated whether children with amblyopia have increased risk of cardiometabolic disorders in adult life.

**Methods:**

This was a cross-sectional and longitudinal analysis of 126,399 United Kingdom Biobank cohort participants who underwent ocular examination. A subset of 67,321 of these received retinal imaging. Data analysis was conducted between November 1st 2021 and October 15th 2022. Our primary objective was to investigate the association between amblyopia and a number of components of metabolic syndrome and individual cardiometabolic diseases. Childhood amblyopia, dichotomised as *resolved* or *persisting* by adulthood, cardiometabolic disease and mortality were defined using ophthalmic assessment, self-reported, hospital admissions and death records. Morphological features of the optic nerve and retinal vasculature and sublayers were extracted from retinal photography and optical coherence tomography. Associations between amblyopia and cardiometabolic disorders as well as retinal markers were investigated in multivariable-adjusted regression models.

**Findings:**

Individuals with persisting amblyopia (n = 2647) were more likely to be obese (adjusted odds ratio (95% confidence interval): 1.16 (1.05; 1.28)), hypertensive (1.25 (1.13; 1.38)) and diabetic (1.29 (1.04; 1.59)) than individuals without amblyopia (controls, (n = 18,481)). Amblyopia was also associated with an increased risk of myocardial infarction (adjusted hazard ratio: 1.38 (1.11; 1.72)) and death (1.36 (1.15; 1.60)). On retinal imaging, amblyopic eyes had significantly increased venular caliber (0.29 units (0.21; 0.36)), increased tortuosity (0.11 units (0.03; 0.19)), but lower fractal dimension (−0.23 units (−0.30; −0.16)) and thinner ganglion cell-inner plexiform layer (mGC-IPL, −2.85 microns (−3.47; −2.22)). Unaffected fellow eyes of individuals with amblyopia also had significantly lower retinal fractal dimension (−0.08 units (−0.15; −0.01)) and thinner mGC-IPL (−1.14 microns (−1.74; −0.54)). Amblyopic eyes with a persisting visual deficit had smaller optic nerve disc height (−0.17 units (−0.25; −0.08)) and width (−0.13 units (−0.21; −0.04)) compared to control eyes.

**Interpretation:**

Although further research is needed to understand the basis of the observed associations, healthcare professionals should be cognisant of greater cardiometabolic dysfunction in adults who had childhood amblyopia. Differences in retinal features in both the amblyopic eye and the unaffected non-amblyopic suggest generalised versus local processes.

**Funding:**

10.13039/501100000265Medical Research Council (MR/T000953/1) and the 10.13039/501100000272National Institute for Health and Care Research.

**Research in context**

**Evidence before this study**

We searched Pubmed for articles from inception to November 11th, 2023, with the search terms “amblyopia”, and “cardio∗”, “cerebro∗, and metabol∗, applying no language restrictions. Investigations into the longer-term impact of childhood amblyopia predominantly focused on psychosocial factors. While associations between childhood amblyopia, its treatment and adverse self-reported mental wellbeing in adulthood were consistent across several prospective cohort studies, evidence of any difference in educational, employment or economic attainment was conflicting. Adults with childhood amblyopia were found to self-report poorer physical wellbeing in one observational study. There was no published investigation of the association between non-communicable disease burden and amblyopia.

**Added value of this study**

In this large United Kingdom-based prospective cohort study, adults who had amblyopia in childhood were more likely to have hypertension, obesity, and metabolic syndrome in adulthood as well as an increased risk of heart attack. Even those with unilateral amblyopia had bilateral retinal morphological differences from those without amblyopia suggesting generalised versus local disease processes. Those with persisting (reduced visual acuity) disease exhibited abnormal optic nerve morphology in contrast to those with resolved (normal visual acuity) amblyopia.

**Implications of all the available evidence**

Adults who had amblyopia in childhood have increased risk of cardiovascular disease and metabolic dysfunction. Optic nerve morphology should be investigated further as a prognostic factor for treatment response in children undergoing treatment for amblyopia. As a leading cause of childhood visual impairment, amblyopia may also represent a relatively common and accessible neurodevelopmental model for research into the early life factors of health and disease.

## Introduction

Nobel-prize winning research has long suggested amblyopia (“lazy eye”) as a great model of human neuroplasticity and neurodevelopment.^1^ Classically unilateral, primary amblyopia affects 1–3% of children globally^2^ and is characterised by aberrant competitive interaction between the cortical afferents of the two eyes.^3^ Despite considerable improvements in visual outcomes owing to childhood population screening programs and timely ophthalmic intervention (e.g. refractive correction or optical penalisation of the contralateral eye), many individuals develop longstanding monocular visual impairment, which persists into adulthood (*persisting* unilateral amblyopia).^4^, ^5^, ^6^ The relationship between the intrauterine environment, neurodevelopment, and non-communicable disease (NCD) in later life has been actively investigated since the 1990s, initially focusing on early environmental risk factors and ischaemic heart disease,^7^ and more recently the association between neurodevelopment and cardiometabolic syndrome.^8^^,^^9^ Amblyopia has also been directly and indirectly, through ocular risk factors of strabismus and refractive error, linked with adverse parent-origin factors, including increased maternal age, maternal smoking, alcohol consumption and lower socioeconomic status.^10^, ^11^, ^12^, ^13^, ^14^ There are consistent associations between these perinatal risk factors and cardiometabolic disease in adulthood.^15^^,^^16^ Furthermore, the apparently unaffected ‘normal’ eyes of individuals with amblyopia have retinal morphological differences, pointing to generalised versus localised systemic structural dysregulation of brain and visual pathways in amblyopia.^17^ Despite its widely recognised importance to national screening policies,^6^ research on the longer term and broader impacts of living with persisting amblyopia into adult life has been limited. Poorer overall general and mental health, and wellbeing have been reported,^4^ but amblyopia associations with cardiometabolic disorders have not previously been systematically investigated.

Drawing together the evidence on the early life influences on neuro-development (including childhood amblyopia specifically) and on cardiometabolic disorders of adult, with the evidence on poorer long term (adult) health of those with amblyopia and the emerging evidence on the relationship between neuro-development and cardiometabolic syndrome, we undertook the current study. Using a multimodal approach we investigated *whether* individuals with childhood amblyopia have different odds of cardiometabolic disorders in later life, compared to non-amblyopes.

## Methods

### Participants and data collection

This was a cross-sectional and longitudinal analysis from 126,399 United Kingdom Biobank (UKBB) participants, aged 40 years or older and recruited between 2006 and 2010, with visual acuity, refractive error measured and other ophthalmic assessments available in both eyes (Supplementary Figure S1). The analysis was conducted between November 1st 2021 and October 15th 2022. Participants who failed the ophthalmic examination, lacked bilateral ophthalmic data, had eye surgery within 4 weeks prior to the examination, had eye disease, lacked of amblyogenic factors, or had bilateral amblyopia were excluded from the analysis. Detailed health data for the participants were collected using a combination of physical measurements, biological assays and longitudinal linkage of multiple health record systems, particularly Hospital Episode Statistics (HES), United Kingdom's National Health Service health administrative data set and touch screen questionnaires capturing general chronic diseases and eye conditions, including amblyopia and any previous treatments. A subset of 67,321 of these participants also had colour fundus photography (CFP) and optical coherence tomography (OCT). We utilised the baseline data collected from 2006 to 2010 supplemented by the subsequent cycles of data collection until 2020. More information on the enhanced ophthalmic examination, other physical assessments, and biological samples are available at the UKBB website (https://www.ukbiobank.ac.uk/). Our objectives were firstly to assess whether individuals with unilateral amblyopia were more likely than those without amblyopia to have cardiovascular disease and metabolic syndrome. To examine whether any association could be mediated through visual acuity, we also compared independently those with resolved (normal visual acuity) and those with persisting amblyopia against non-amblyopes. Secondly, we sought to investigate whether affected and unaffected fellow eyes of these individuals had retinal and optic nerve morphological differences on in-vivo imaging compared to the eyes of healthy controls.

### Classification of amblyopia

Through touch screen questionnaires capturing general chronic diseases and eye conditions, including prior childhood amblyopia and any previous treatments, participants were asked, during recruitment at the assessment centres, whether they were treated for amblyopia (‘lazy eye’) in childhood. Using our previously validated hierarchical approach,^4^ participants were classified as having amblyopia if they self-reported amblyopia or treatment with any of the following corroborating evidence: (1) strabismus, (2) significant anisometropia (difference of at least −1.00 dioptre [D]/+1.00 D between eyes), (3) significant astigmatism (cylinder power ≥1.00 D), (4) significant refractive error per se (i.e. −3.00 D/+3.00 D or more extreme), (5) less severe refractive error but visual impairment without any other underlying eye disease (such as stimulus deprivation amblyopia or cataract), and (6) current emmetropia (absence of refractive error, −0.99 D to +0.99 D) in participants who self-reported hypermetropia correction in childhood and at least mild visual impairment and no other eye disease. Amblyopia was also identified in participants through record linkage to relevant treatment codes using HES data. Those with amblyopia were dichotomised as “resolved” (current normal or near normal, better than 0.06 logarithm of the minimum angle of resolution [logMAR]) or “persisting” (residual acuity deficit despite treatment in childhood, including visual impairment/blindness, but excluding bilateral amblyopia) amblyopia. Participants with bilateral normal visual acuity (i.e. 0.0 logMAR) and without primary refractive error (i.e. emmetropic) or any other eye disease or amblyogenic factors (using self–report, ophthalmic examination and HES data), were the comparator group (‘controls’).

### Cardiometabolic disorder ‘outcomes’

Cardiovascular outcomes were collected from the self-reported touchscreen questionnaire on diagnosis by a doctor of i) diabetes (UKBB field “2443”), ii) high blood pressure (derived from field “6150”), and iii) cardio/cerebrovascular disease, namely angina, heart attack, stroke (all derived from field “6150”). Participants’ body weight and height were measured using standard procedures during the initial assessment, and body mass index (BMI, kg/m^2^) was calculated. Obesity was defined as BMI >30 kg/m^2^. The co-existence of diabetes, high blood pressure and obesity was used to define the presence of metabolic syndrome.^18^ Incident myocardial infarction (MI, field “42,000”) and all-cause stroke (field “42,006”) were derived from UKBB algorithmically-defined outcomes, a standardised approach for identifying the earliest recorded date of a given health outcome based on death records, hospital admissions data and self-report.^19^^,^^20^ Mortality date was attained through linkage with national death registry data.

### Ocular morphology ‘outcomes’

Macula-centred 45-degree colour fundus photography (CFP) and optical coherence tomography (OCT) were acquired using the Topcon 3D-OCT 1000 MKII (Topcon Corporation, Tokyo, Japan).^21^^,^^22^ OCTs covered a 6.0 mm × 6.0 mm area and had 128 horizontal B scans and 512 A scans per B scan. Retinal vasculature features, comprising arteriolar and venular caliber, fractal dimension, distance tortuosity and vessel density, and optic nerve cup-disc ratio [CDR]) were extracted using the open-source segmentation and deep learning-based pipeline, AutoMorph^23^ (Supplementary Figure S2) which has been extensively validated across multinational datasets. OCTs were segmented using the Topcon Advanced Boundary Segmentation Tool (TABS, version 1.6.2.6), leveraging dual-scale gradient information for automated segmentation of retinal sublayers.^22^ Retinal nerve fibre layer (RNFL) and macular ganglion cell-inner plexiform layer (mGC-IPL) thickness were defined according to the International Nomenclature for OCT panel^24^ (Supplementary Figure S2). Retinal sublayers for the four parafoveal subfields defined in the Early Treatment Diabetic Retinopathy Study, were averaged for analysis.^25^ For image quality control, we excluded images classified as poor quality by the AutoMorph image quality module and the most extreme 10% of OCT images based on specific image quality metadata generated by TABS for each scan, as per previous reports in the UKBB study.^22^

For the retinal imaging analysis, we included individuals with valid eye data with either confirmed amblyopia (cases) or no amblyopia and no amblyogenic factors (controls) but with no other eye disease (Supplementary Figure S3). Recognising that there may be differences in localised (eye level) or generalised (person level) associations, we firstly compared the affected eye of individuals with *persisting* unilateral amblyopia against one randomly chosen eye of controls. Secondly, we compared the fellow (unaffected/normal) eye of affected individuals with one randomly chosen eye of control individuals.

### Statistics

Descriptive statistics comprise mean or frequencies alongside 95% confidence intervals (CI). Differences in the distribution of age was assessed with the t-test whilst differences in the distribution of demographic (sex, ethnicity, deprivation) and clinical outcomes, between amblyopic and non-amblyopic participants were assessed using the two-proportion z-test.

Our pre-planned primary analysis used logistic regression models (base models), adjusted for age, sex (acquired from central registry at recruitment, but in some cases updated by the participant classified as females/males), ethnic background (white ethnic background/other than white ethnic background), and deprivation (quintiles of Townsend index of deprivation 2011) to investigate the association between amblyopia and number of components of metabolic syndrome (multinomial regression), and individual diseases (binary regression). Estimates are reported as relative risk ratios (RRR) or odds ratios (OR) respectively, alongside 95% CIs. The association between amblyopia and cardiovascular biomarkers (body mass index, systolic and diastolic blood pressure, and glycated haemoglobin) for the base models was also investigated using linear regression models and results are presented as regression coefficients (95% CI). We used propensity score matching, implementing the nearest neighbour algorithm with no caliper, to account for confounding and differences between participants with amblyopia and controls and to estimate treatment effects for the treated (ATET). We matched participants with amblyopia (treated) and participants without (controls) based on age, sex, ethnic background, and deprivation. For investigating the association between amblyopia and time of incident MI, stroke, and death, we estimated hazard ratios (HR) using Cox proportional hazards. The risk period was defined from the initial assessment visit until the earliest of death (censored), clinical event or conclusion of the data coverage period (censored October 11th, 2021). Individuals with a previous MI or stroke were excluded from the respective analysis (Supplementary Table S3). The assumption of proportional hazards was assessed using global and covariate-specific χ^2^ testing and visualisation of graphs of scaled Schoenfeld residuals against transformed time.^26^ Nonlinearity between age and log hazard was assessed by plotting Martingale residuals of the null Cox proportional hazards model.

To investigate the association between persisting unilateral amblyopia and retinal morphology we have also used two binary regression models a) the base model, adjusted as previously for age, sex, ethnic background, deprivation and additionally adjusted for spherical error as this can affect retinal morphological measurements,^27^ and b) the full model additionally adjusted for smoking status, alcohol consumption, hypertension, BMI and diabetes mellitus.^28^ To aid interpretability, retinovascular features were scaled (z-score) for model fitting. All tests were two-sided and the significance level was set to 0.05. Analysis was done on a complete case analysis basis with missing data at each step described. Analyses were performed using Stata version 17.0 (StataCorp. 2021. *Stata Statistical Software: Release 17*. College Station, TX: StataCorp LLC) and R version 4.1.0 (R Core Team, 2021. R Foundation for Statistical Computing, Vienna, Austria).

### Ethics

Ethics Committee approval was obtained for UKBB (ref: 06/MRE08/75). This study adhered to the ethical standards outlined in the Declaration of Helsinki. All participants had given their informed consent.

### Role of funding source

The funders of the study had no role in the study design, data collection, data analysis, data interpretation, or writing of the manuscript.

## Results

### Amblyopia and cardiometabolic disorders

Of the 126,399 UKBB participants with visual acuity and refractive error measured in both eyes, 111,304 had valid measurements in both eyes. Of the latter 3238 (2.9%) were confirmed amblyopes. The clinical sample analysis used data from 21,702 participants with complete ophthalmic and demographic data. Of 3221 participants (14.8%) with confirmed amblyopia, 82.2% (2647) had *persisting* amblyopia (Supplementary Figure S1). Compared to controls, participants with amblyopia were older by about 6 years on average, mainly of white ethnic background (97%), and self-reported higher medically diagnosed disease frequency, with similar distributions between those with resolved and persisting amblyopia (Supplementary Table S1; Supplementary Figure S4).

**Fig. 1:**
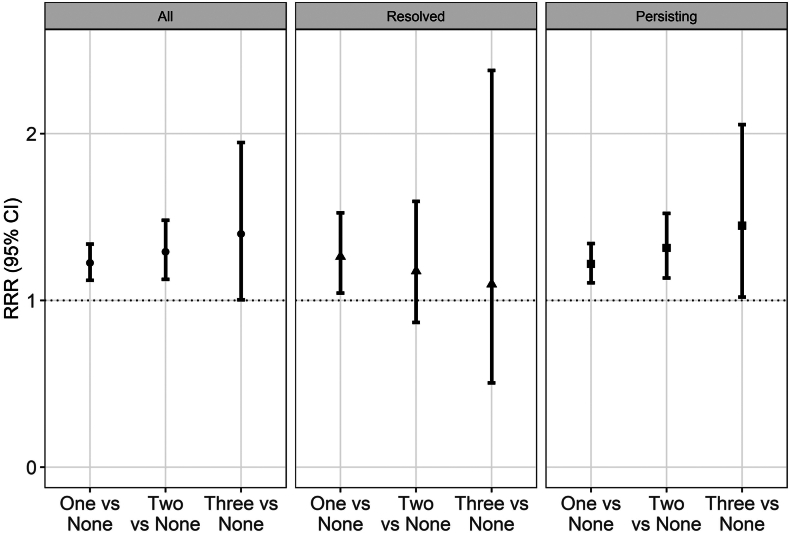
Association between amblyopia category (all, resolved and persisting) and burden of metabolic syndrome (number of components). Legend: Medically diagnosed diabetes mellitus (UK Biobank field “2443”) and high blood pressure (derived from field “6150”), and obesity (classified as BMI>30 kg/m^2^, derived from fields “21,002” for weight and “12,144” for height) were the three components relevant to the presence of metabolic syndrome. CI: Confidence interval; RRR: Relative risk ratio.

**Fig. 2:**
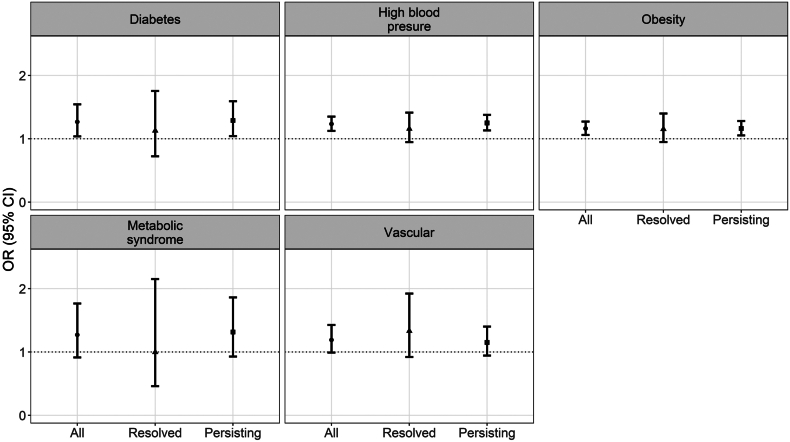
Association between amblyopia category (all, resolved and persisting) and self-report of diseases diagnosed by medical doctor. Legend: Metabolic syndrome was defined according to medical diagnosis for diabetes mellitus (UK Biobank field “2443”) and high blood pressure (derived from field “6150”), and obesity (classified as BMI>30 kg/m^2^, derived from fields “21,002” for weight and “12,144” for height). Medically diagnosed vascular/heart disease is relevant to being diagnosed with heart attack, stroke or angina all derived from field “6150”. CI: Confidence interval; OR: Odds ratio.

In the base models, participants with amblyopia had higher relative risk ratio of one (RRR (95% CI): 1.22 (1.12; 1.34)), two (1.29 (1.13; 1.48)), or three components of metabolic syndrome (1.40 (1.00; 1.95)), but this was driven by the associations with the persisting amblyopia group (Fig. 1; Table 1). Participants with persisting amblyopia had higher odds of diabetes (1.29 (1.04; 1.59)) and high blood pressure (1.25 (1.13; 1.38)) and being classified as obese (1.16 (1.05; 1.28)), but no significant associations with metabolic syndrome overall *per se* were found (Fig. 2; Supplementary Table S2). Overall amblyopia was also associated with higher odds of diagnosis of vascular problems and previous heart attack. Adjusted analyses of quantitative traits confirmed the positive association of persisting amblyopia with body mass index (0.40 (0.20; 0.60) kg/m^2^), glycated haemoglobin (0.48 (0.24; 0.73) mmol/mol) and systolic (0.83 (0.14; 1.51) mmHg), but not diastolic blood pressure (0.02 (−0.39; 0.44) mmHg) (Table 2). Participants with amblyopia had an increased risk of MI (HR = 1.38 (1.11; 1.72), total follow-up time: 241,187 years, average follow-up time: 11.6 ± 1.4 years), and all-cause death (HR = 1.36 (1.15; 1.60), total follow-up time: 246,237 years, average follow-up time 11.6 ± 1.3 years) but not stroke (1.20 (0.89; 1.62), total follow-up time: 242,840 years, average follow-up time: 11.67 ± 1.2 years, Supplementary Tables S3 and S4).

**Table 1:** Association between classification of amblyopia (i.e. i) all, ii) resolved, iii) persisting) and cardiometabolic diseases diagnosed by medical doctor.

Results are odds ratios (95% confidence intervals)) derived from binary logistic regression models, adjusted for all the covariates shown in table. Metabolic syndrome was not medically diagnosed but was defined as being medically diagnosed with diabetes, high blood pressure and obesity.

**Table 2:** Association between classification of amblyopia (i.e. i) all, ii) resolved, iii) persisting) with cardiometabolic biomarkers.




Results are beta coefficients (95% confidence intervals) derived from linear regression models, adjusted for all the covariates shown in table.

**Fig. 3:**
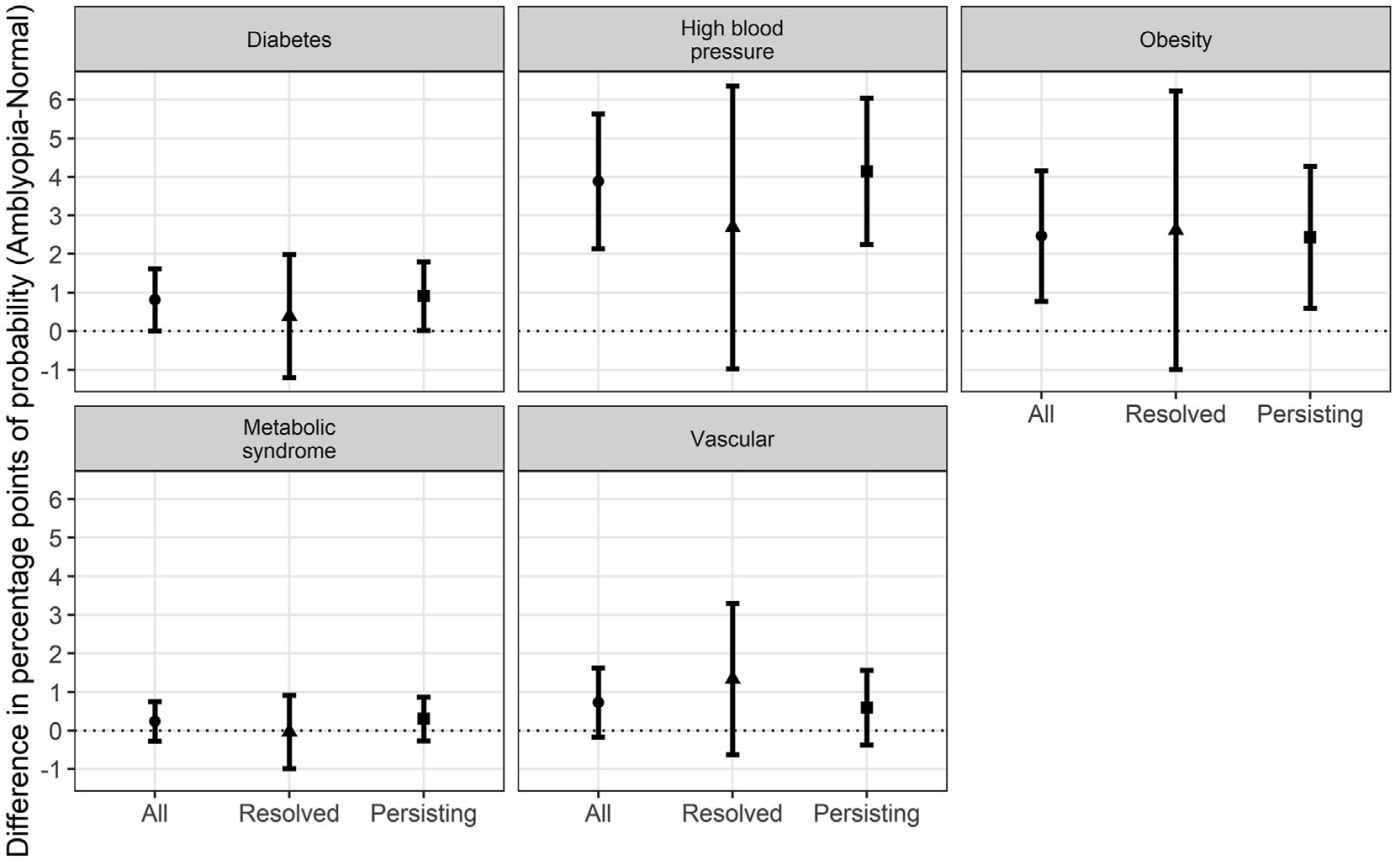
Difference in the probability of self-reporting a disease diagnosed by medical doctor, between participants with amblyopia and controls (without amblyopia), by amblyopia category. Legend: Metabolic syndrome was defined according to medically diagnosed diabetes mellitus (UK Biobank field “2443”) and high blood pressure (derived from field “6150”), and obesity (classified as BMI>30 kg/m^2^, derived from fields “21,002” for weight and “12,144” for height). Medically diagnosed vascular/heart disease is relevant to being diagnosed with heart attack, stroke or angina all derived from field “6150”.

Results from the propensity score matching (to account for the observed differences in age and ethnic background and marginal differences in sex and deprivation between the amblyopia and control groups, Supplementary Table S5) also showed that participants with amblyopia were more likely to have medically diagnosed high blood pressure or obesity compared to controls (Fig. 3).

Adjustment for centre within the analyses did not alter the findings (data not shown).

### Amblyopia as retinal morphology

For the retinal imaging analysis, 35,061 controls (35,061 control eyes), and 831 individuals with unilateral amblyopia were included (Supplementary Figure S2). Individuals with amblyopia were older and more likely to be of white ethnicity. Compared to control eyes, affected eyes had significantly thinner inner retinal layers (mRNFL and mGC-IPL) and several retinovascular differences, as shown in Supplementary Table S6. The optic nerve height of amblyopic eyes was also smaller than control eyes. The fellow (unaffected) eyes of individuals with persisting unilateral amblyopia also exhibited similar differences to unaffected control eyes (Supplementary Table S6).

On the fully adjusted models, amblyopic eyes had significantly increased venular caliber, distance tortuosity and lower fractal dimension compared to unaffected eyes of participants with unilateral amblyopia (Table 3). They also had thinner mGC-IPL (−2.85 (−3.47; −2.22) microns). Affected eyes had reduced optic disc height and disc width compared to control eyes. The unaffected fellow eyes of those with amblyopia were also different to controls, with lower retinal fractal dimension (−0.08 (−0.15; −0.01) per SD) and thinner mGC-IPL (−1.14 (−1.74; −0.54) microns) (Table 3). Specifically, affected eyes of those with either “persisting” or “resolved” amblyopia had wider venular caliber and thinner mGC-IPL. However, smaller optic nerve disc height and width differences were only statistically significant for “persisting” amblyopic eyes (Supplementary Table S7).

**Table 3:** Differences in retinal morphology between affected and unaffected (fellow) eyes of individuals with amblyopia compared to controls.

Results are beta coefficients (95% confidence intervals) derived from linear regression. Base model was adjusted for age, sex, ethnicity, socioeconomic deprivation and refractive error. Full model was the base model additionally adjusted for hypertension, diabetes mellitus, alcohol consumption, smoking history, and body mass index. mGC-IPL: macular ganglion cell-inner plexiform layer, mRFNL: macular retinal nerve fiber layer, SD: standard deviation.

## Discussion

From a large epidemiological study of British adults, we report that individuals with persisting childhood amblyopia (vision deficit despite treatment) are more likely to be diagnosed with cardiometabolic disorders later in adult life, compared to those without amblyopia and independent of socio-demographic risk factors. These individuals also have retinal morphological changes previously reported in association with cardiovascular and metabolic disease.^28^, ^29^, ^30^ Retinal biomarker differences are evident in both the affected amblyopic eye and the putatively ‘normal’ fellow eye in those with persisting amblyopia, but not with amblyopia that resolved following treatment. Together these findings suggest generalised early life neurodevelopmental dysregulation (here captured in a common neurodevelopmental disorder) is associated with cardiometabolic conditions in later life.

The UKBB is unique in combining retinal imaging, detailed ophthalmic and general medical and health data for a very large number of people, and the power to investigate the relationship between amblyopia and long-term clinical outcomes. Notwithstanding these key strengths, there are some limitations and contextual issues to consider in interpretation of our results. UKBB participants overall are less obese and have healthier lifestyles,^31^ although the risk factor associations estimated from UKBB do generalise well to nationwide registry and survey data for England and Scotland.^32^ Furthermore in our previous report^4^ investigating broader functional outcomes in individuals with persisting unilateral amblyopia in UKBB, we observed associations consistent with other prospective cohort studies.^5^^,^^33^^,^^34^ At the cost of reducing our potential sample size by excluding a proportion of the original cohort due to coexistence of other eye conditions, our hierarchical approach for defining amblyopia and amblyogenic risk factors integrating ophthalmic and other physical measurements, national health service data and self-reported medically diagnosed outcomes minimises the potential recall and misclassification bias in relation to both amblyopia status and non-communicable diseases and reduces potential confounding. Our strategy yields estimates of amblyopia prevalence comparable to reports from other British population-based analyses.^5^^,^^35^ Among possible limitations, whilst our sample of participants with amblyopia was large, it comprised predominantly those with *persisting* amblyopia (reflecting outcomes from prevailing treatment at least four decades ago). Our primary analysis used self-reported medical conditions which are subject to recall bias however these were medically diagnosed and, moreover, our secondary analyses investigating physical assessment (e.g. body mass index) and biochemical data (e.g. glycated haemoglobin) showed consistent results. As with any observational study, residual confounding is possible, even after adjusting for all potential confounders. Similarly, our study design and analysis cannot interrogate any causal relationships between amblyopia and cardiometabolic outcomes.

We report for the first time, to our knowledge, that individuals with childhood amblyopia who have a residual acuity deficit (‘persisting’ amblyopia) are more likely than those who never had amblyopia to be diagnosed in adult life with diabetes mellitus, obesity, and hypertension and are at higher risk for myocardial infarction. While there were significant associations with individual metabolic syndrome components, this was not the case for metabolic syndrome overall. We suspect this may be due to the low prevalence of metabolic syndrome in this population-based cohort (0.9% in controls, 1.6% in affected) as effect estimates for this association were even larger but with wider precision estimates. We did not assess for incident diabetes mellitus as previous validity studies have shown that secondary care data can miss nearly half of such cases.^36^ However, investigating incident metabolic dysfunction in individuals with amblyopia would be informative future research, given the cross-sectional associations we report. There are no studies to which we can directly compare our findings as population-based research on the health of individuals with amblyopia is limited.^4^^,^^5^ In a smaller sample from the 1958 British Birth Cohort with amblyopia (51 individuals with moderate/severe amblyopia), there were no statistically significant differences in likelihood of reporting poor health conditional on amblyopia status,^5^ although participants were assessed at a younger age when most would not yet have developed cardiometabolic disorders. By contrast, our prior study of the broader functional impacts and well-being outcomes in adult life in UKBB (age range 40–69 years), found persisting unilateral amblyopia was associated with adverse self-assessed general health.^4^ The present study offers some possible explanations for that observation.

What might explain the observed associations with amblyopia in the present study? Although children with amblyopia may have worse motor skills and reading speed,^37^^,^^38^ this does not seem to significantly affect their educational and employment attainment nor economic outcomes and socioeconomic status, all important to cardiometabolic health.^4^^,^^5^ Whether the reported poorer mental health and emotional wellbeing associated with amblyopia and its treatment in some studies translates into potentially harmful lifestyle behaviours, such as a more sedentary behaviour, is unknown.^4^^,^^39^ Notably, we found no differences in smoking status or alcohol consumption between those with amblyopia and those without. Also, the significant differences in retinal morphology persisted even after adjustment for these and other key cardiometabolic risk factors. Although our study cannot explain what underlines these association, our results align well with existing evidence on the developmental origins of adult disease.^40^ One possible mechanism is the impact on both the neurodevelopment and the cardiometabolic axes of a suboptimal intrauterine environment, manifesting especially as intra-uterine growth restriction (Supplementary Figure S6). Our findings suggest that amblyopia may be usefully considered among other neurodevelopmental outcomes affected by early life factors and indeed future work may also consider investigating the relationship between other forms of neurodevelopmental impairment and adult cardiometabolic dysfunction in a suitably large and representative sample of individuals with and without amblyopia. Notably many of the ‘intermediate’ ophthalmic characteristics associated with risk of amblyopia, such as refractive error or strabismus, are more common amongst children born pre-term and/or of low birthweight. The absence of accurate perinatal data (e.g. birthweight data was missing in 48% of participants) and risk of recall bias precluded us from using the available perinatal data or reliably imputing it. Nevertheless, our findings align with a recent study using mendelian randomization to postulate a causal link between low birthweight and self-reported amblyopia.^41^ However, the current study design cannot address questions of causality and further work is needed to explore the mechanistic aspects of this novel association.

We analysed retinal morphological features in a subset who underwent enhanced ophthalmic assessment with retinal imaging. The striking differences in retinal morphological biomarkers of cardiometabolic disorders in individuals with and without amblyopia are consistent with our previous understanding of these disorders. Their existence in both affected and unaffected fellow eye points to generalised versus localised changes in microvascular and central nervous systems. To contextualize our findings, the adjusted mGC-IPL thickness difference in the affected (−2.85 microns) and unaffected fellow (−1.14 microns) eye was equivalent to eighteen and eight years of older age, respectively, using previous estimates from UKBB data.^28^ Differences in retinal morphology between the two eyes in people with amblyopia have been identified from childhood.^17^ However a recent meta-analysis of OCT angiography concluded that while amblyopic eyes have significantly different vessel density compared to healthy controls, there was no difference between affected and unaffected fellow eyes.^42^ By contrast, individuals with incipient metabolic syndrome and cardiovascular disease exhibit thinner mGC-IPL,^43^ corroborating our findings of increased HbA1c and risk of myocardial infarction in those with amblyopia. Additionally, retinal fractal dimension was significantly lower in both eyes of individuals with unilateral amblyopia and is negatively associated with hypertension and cardiovascular disease.^30^ Longitudinal studies of the evolution of the various retinal morphological features would help to determine the role of degenerative mechanisms.

Optic disc size is known to vary with age, sex, and refractive error.^44^ Adjusting for these and other factors, we found amblyopic eyes with persisting visual deficit exhibited significantly smaller optic disc height and width compared to healthy controls whereas ‘recovered’ amblyopic eyes (i.e. normal visual acuity) were not different to control eyes. The present study cannot establish if the structural differences represent an underlying primary cause of amblyopia (and a potential prognostic marker for treatment success) or indicate an established structural change and visual deficit arising from non-response to treatment carried through into adult life. Although foveal abnormalities of individuals with amblyopia are bilateral and symmetrical regardless of interocular differences in visual acuity,^17^ our evidence suggests this is not the case for the optic nerve. Asymmetric neurodevelopment secondary to gestational insult has been proposed as a cause of asymmetric optic nerve hypoplasia and impaired binocular interactions, manifesting as amblyopia.^45^ Major risk factors for amblyopia, including strabismus and anisometropia, are also not typically present in a persistent form from birth and may result secondary to a primary retinal structural abnormality.^17^ Optic nerve imaging and morphology in amblyopia, aided by newer measurement algorithms may help discriminate between children likely to experience good visual recovery.

Understanding the mechanism(s) that underlie our findings of increased risk of cardiometabolic disorders in adulthood amongst people treated for amblyopia in childhood, will require further and longitudinal research which could yield wide benefits. In the meantime, healthcare professionals should be cognisant that a diagnosis of amblyopia in a child is associated with increased cardiometabolic morbidity in later life.

## Contributors

All authors participated in the study design and approval of the study concept. SKW and VB accessed and verified the raw data and analysed the data. SKW, VB, PH and JSR prepared the initial manuscript. All authors participated in interpretation of data, discussion, and revision of the manuscript. All authors had full access to the data in the study and accept responsibility to submit for publication. JSR had final responsibility for the decision to submit for publication. All authors read and approved the final version of the manuscript.

## Data sharing statement

Data from the United Kingdom Biobank is available to approved researchers upon application. Further information is available at https://www.ukbiobank.ac.uk/.

## Declaration of interests

We declare no competing interests.

## Appendix A Supplementary data

Amblyopia supplement

UKBB Membership
