# Supplementary material for: Associations between unilateral amblyopia in childhood and cardiometabolic disorders in adult life: a cross-sectional and longitudinal analysis of the UK Biobank
Source: eClinicalMedicine. 2024 Mar 7;70:102493. doi: 10.1016/j.eclinm.2024.102493 (PMC11056416; doi:10.1016/j.eclinm.2024.102493)
Supplement: Amblyopia supplement [file mmc1.pdf]

On-line supplementary material

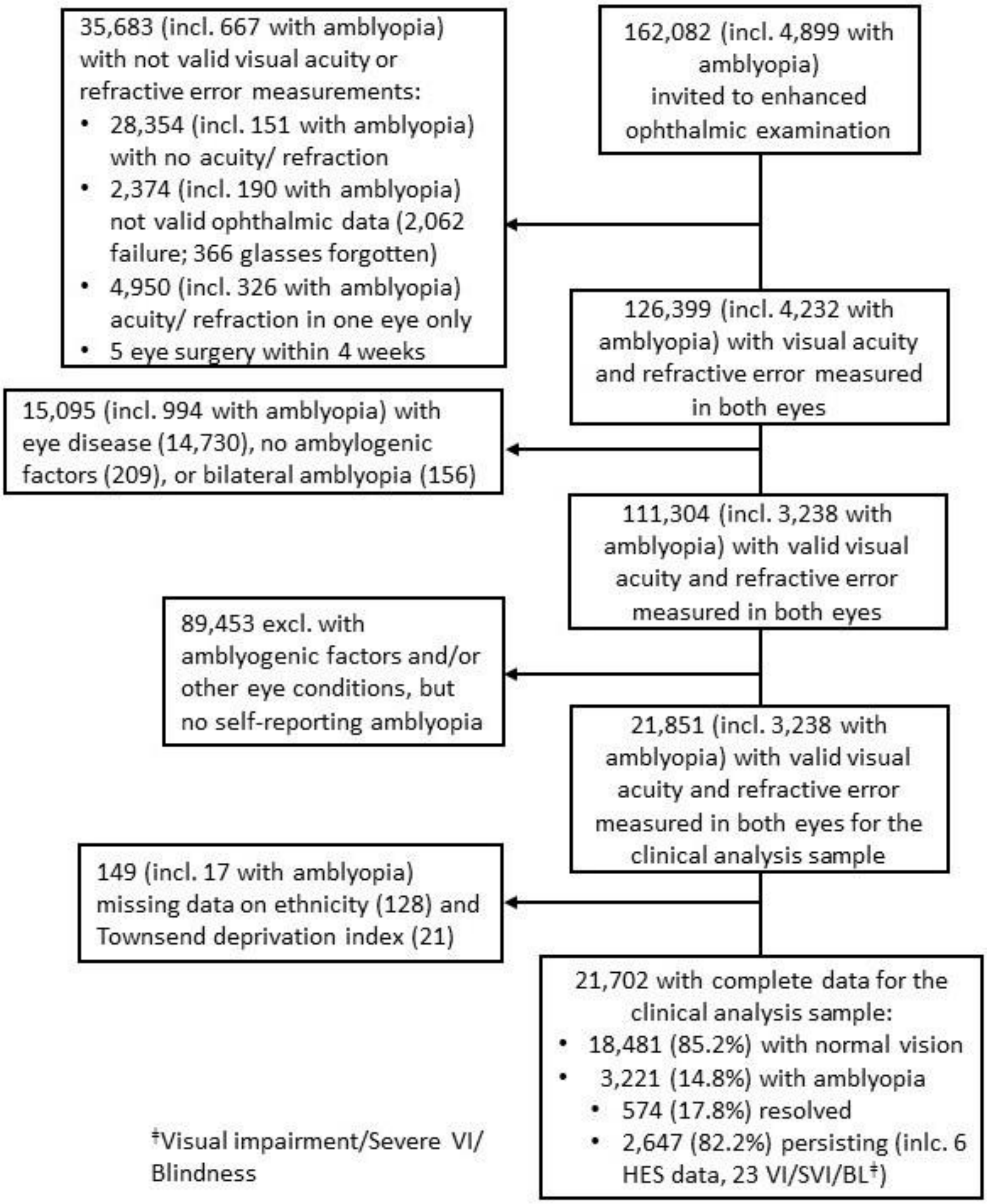

Supplementary Figure 1. Flow of participants in the clinical sample study.

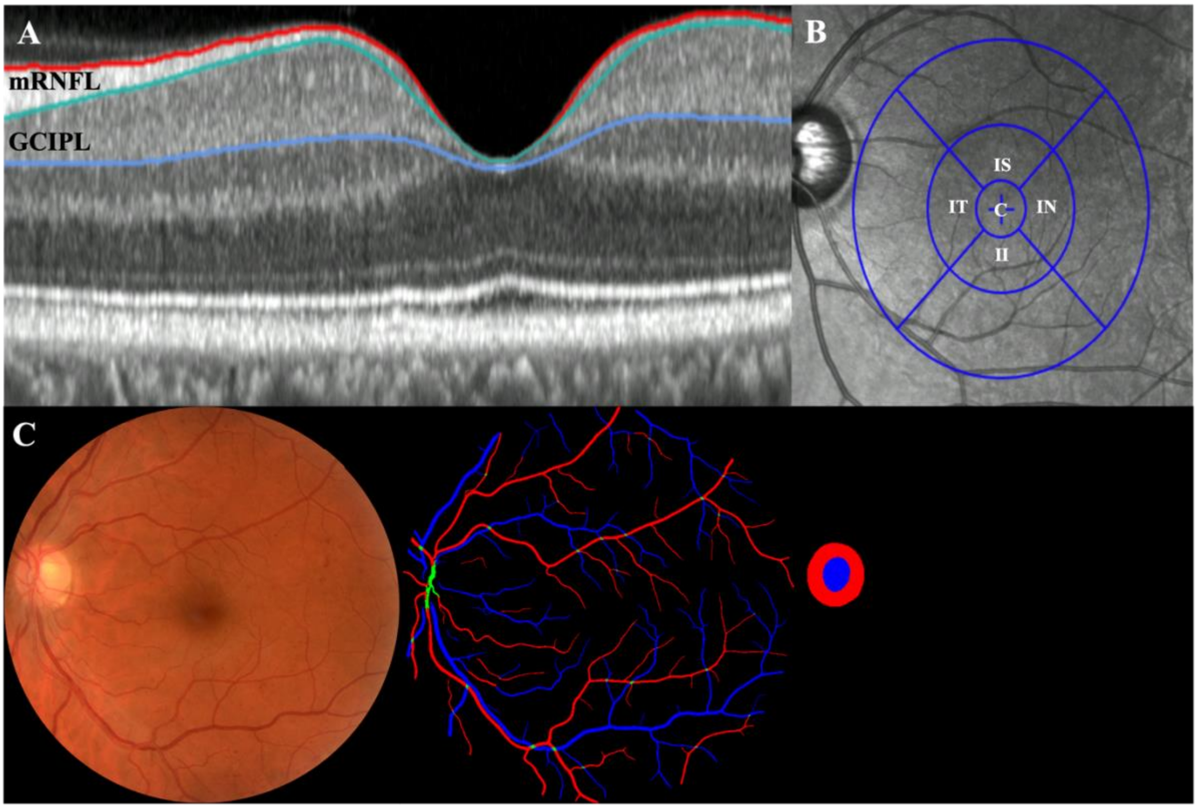

Supplementary Figure 2. Example retinal images. Macula-centered optical coherence tomography with the retinal nerve fiber (mRNFL) and ganglion cell-inner plexiform layer indicated (mGC-IPL) (A). Sublayer thicknesses were averaged across the four parafoveal grids (IS - Inner Superior region; IN - Inner Nasal region; II - Inner Inferior region; IT - Inner Temporal Region) (B). Retinal vasculature and the optic nerve were segmented from raw color fundus photographs by AutoMorph to derive vessel maps, from which morphometric indices were estimated (C).

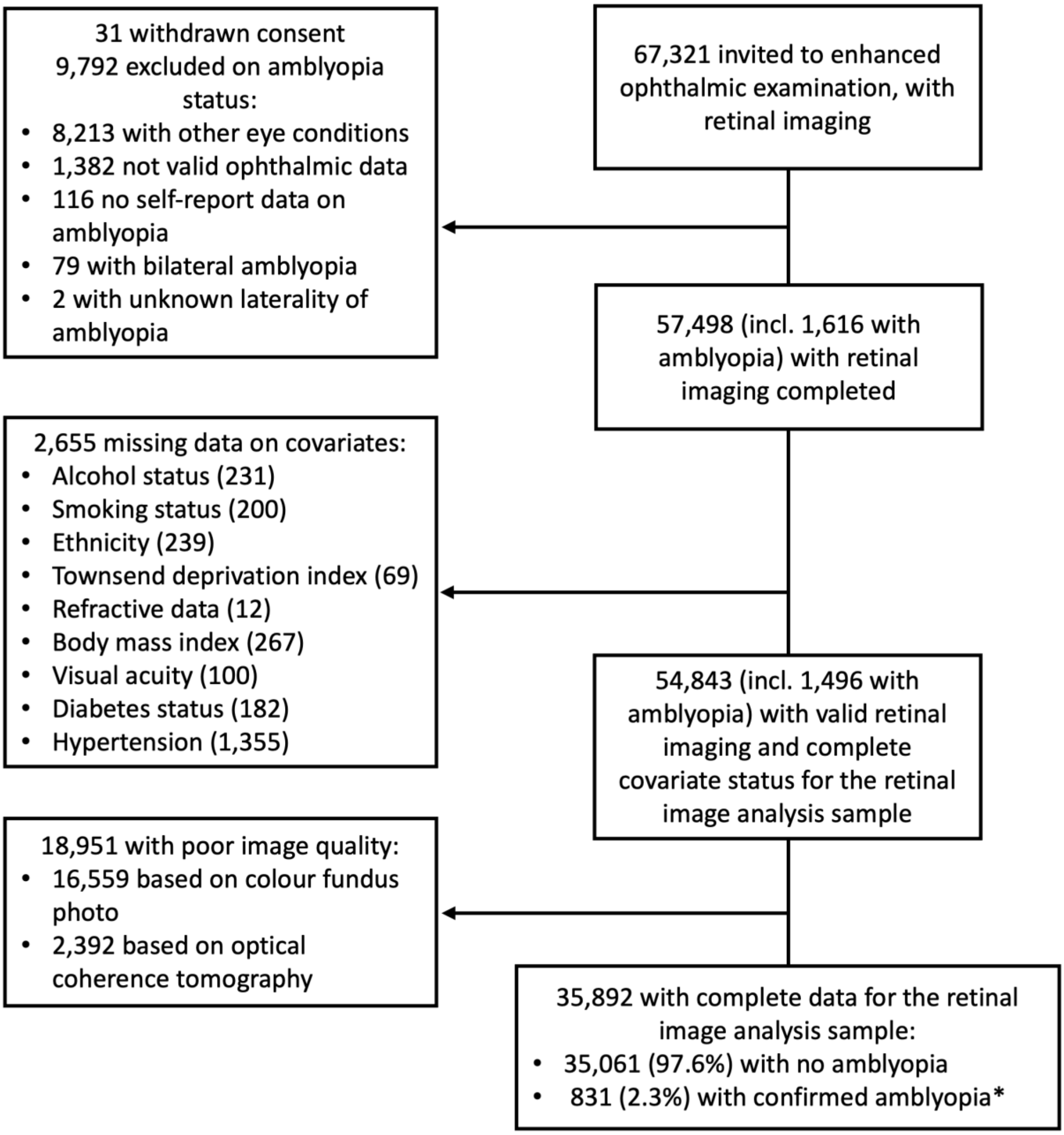

Supplementary Figure 3. Flow of participants in the retinal image analysis.

*From 831 participants with confirmed amblyopia, 623 eyes from 623 participants had sufficient quality retinal imaging of an affected amblyopic eye sufficient for analysis (483 persisting and 140 resolved). 663 eyes from 663 participants were unaffected fellow eyes. The difference is due to image quality control.

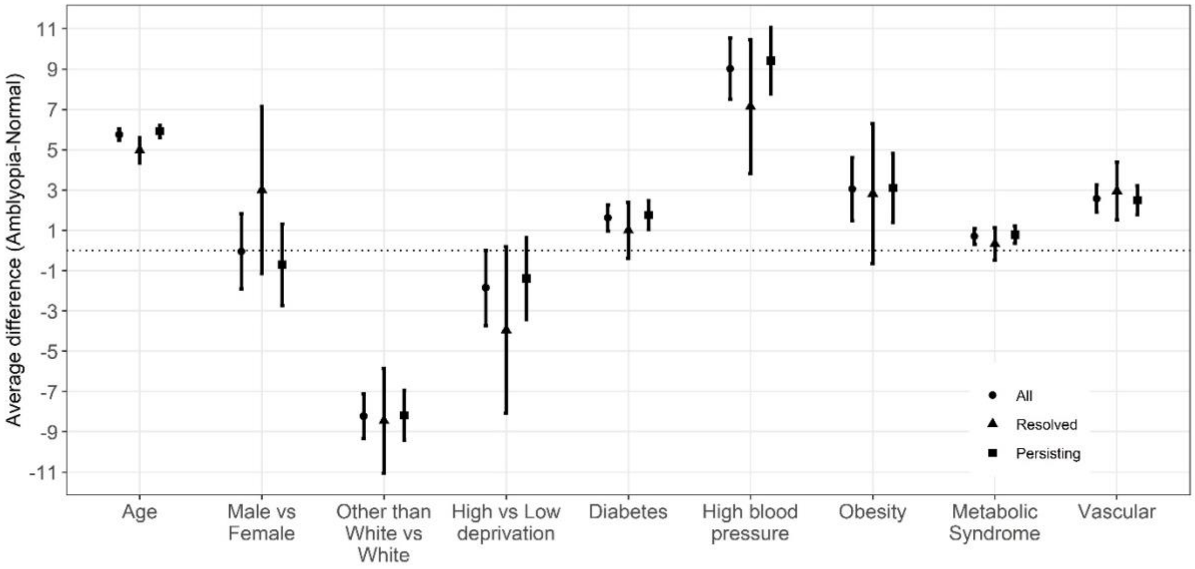

Supplementary Figure 4. Differences in the distribution of demographic and clinical characteristics between participants with amblyopia and unaffected vision, by amblyopia status.

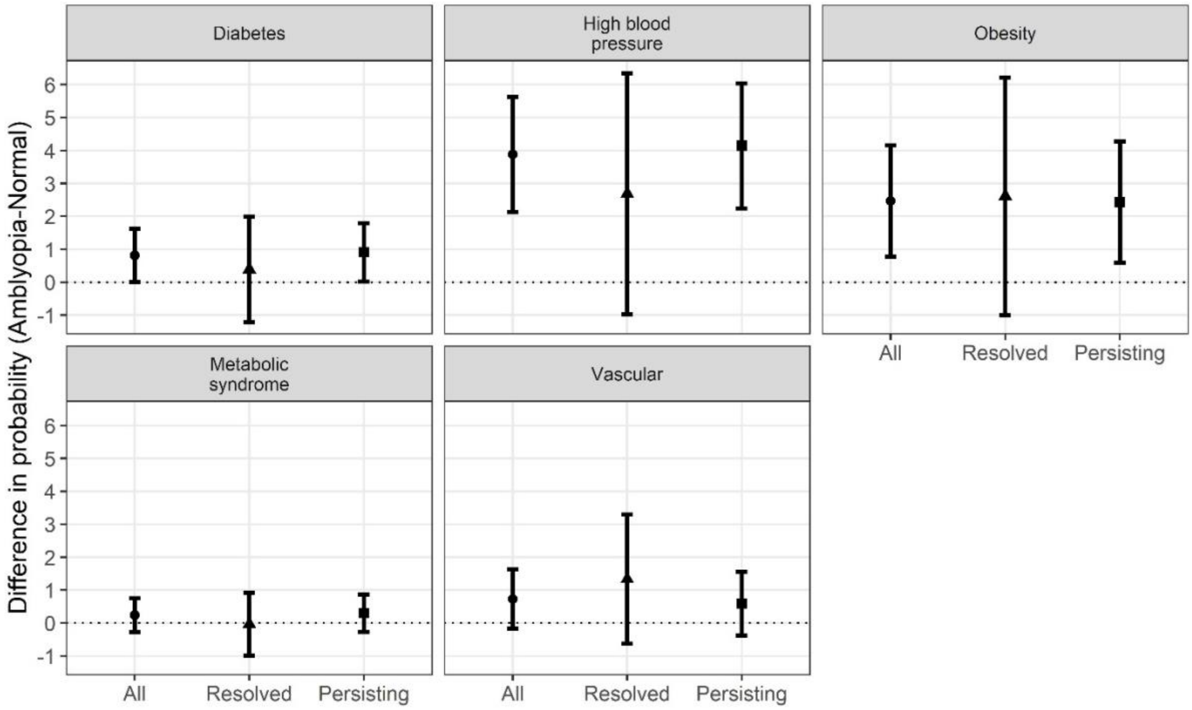

Supplementary Figure 5. Difference in the probability of self-reporting a disease diagnosed by medical doctor, between participants with amblyopia and controls (without amblyopia), by amblyopia category.

Legend: Metabolic syndrome was defined according to medically diagnosed diabetes mellitus (UK Biobank field "2443") and high blood pressure (derived from field "6150"), and obesity (classified as BMI>30 kg/m^2, derived from fields "21002" for weight and "12144" for height). Medically diagnosed vascular/ heart disease is relevant to being diagnosed with heart attack, stroke or angina all derived from field "6150".

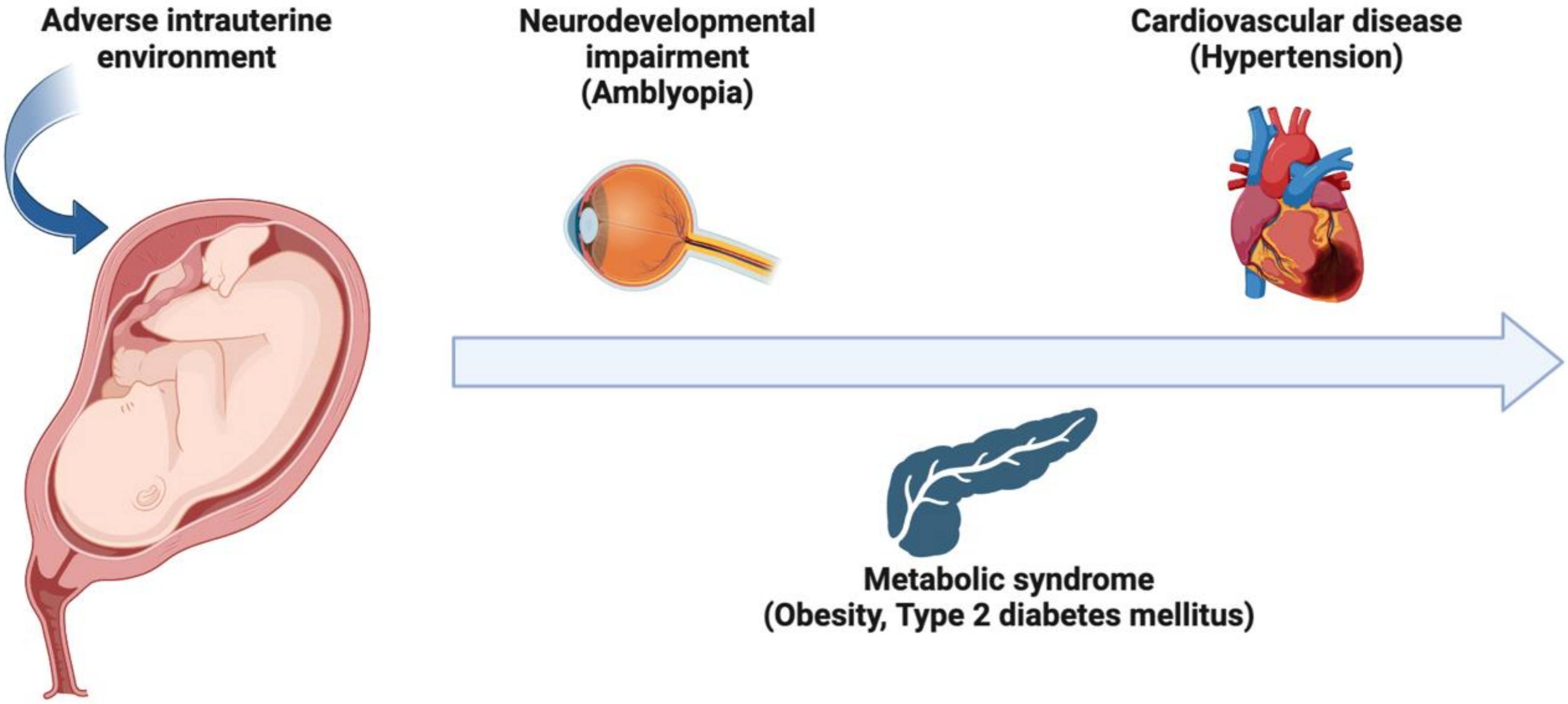

Supplementary Figure 6. Fetal origins of adult disease as pertains to amblyopia. Harmful intrauterine influences result in an increased risk of neurodevelopment impairment, such as amblyopia, in early life as well as cardiometabolic dysfunction manifesting in adulthood.

Supplementary Table 1. Participants' demographic and clinical characteristics by vision status.

	Control (unaffected vision)	Amblyopia		
		All	Resolved	Persisting
n (%)	18,481 (84.6)	3,377 (15.4)	627 (18.6)	2,750 (81.4)
Age at recruitment	51.3	57.1	56.3	57.3
	(51.2; 51.4)	(56.8; 57.4)	(55.7; 57)	(57.0; 57.5)
Male participants, %	47.2	47.1	50.2	46.5
	(46.5; 47.9)	(45.4; 48.9)	(46.1; 54.3)	(44.6; 48.4)
Other than white ethnic background, %	11.2	3.0	2.8	3.1
	(10.8; 11.7)	(2.4; 3.6)	(1.4; 4.1)	(2.4; 3.7)
Live in a highly deprived area, %	46.3	44.4	42.3	44.9
	(45.6; 47.0)	(42.7; 46.1)	(38.3; 46.4)	(43.0; 46.8)
Obesity (BMI >30kg/m^2), %	22.3	25.4	25.1	25.4
	(21.7; 22.9)	(23.9; 26.9)	(21.6; 28.7)	(23.8; 27.1)
Missing, n (%)	*79 (0.43)*	*8 (0.25)*	*1 (0.17)*	*7 (0.26)*
Conditions diagnosed by medical doctor				
Diabetes mellitus, %	2.8	4.5	3.8	4.6
	(2.6; 3.1)	(3.7; 5.2)	(2.3; 5.4)	(3.8; 5.4)
Missing, n (%)	*72 (0.39)*	*8 (0.25)*	*0 (0.0)*	*8 (0.30)*
High blood pressure, %	19.0	28.0	26.2	28.4

		(18.5; 19.6)	(26.5; 29.6)	(22.5; 29.8)	(26.7; 30.2)
	Missing, n (%)	*332 (1.8)*	*94 (2.9)*	*20 (3.5)*	*74 (2.8)*
	Vascular disease, %	3.0	5.6	5.9	5.5
		(2.7; 3.2)	(4.8; 6.4)	(4.0; 7.9)	(4.6; 6.4)
	Missing, n (%)	*48 (0.26)*	*5 (0.16)*	*2 (0.35)*	*3 (0.11)*
Metabolic Syndrome, %		0.9	1.6	1.3	1.7
		(0.8; 1.1)	(1.2; 2.1)	(0.3; 2.2)	(1.2; 2.2)
	Missing, n (%)	*457 (2.5)*	*110 (3.4)*	*21 (3.7)*	*89 (3.4)*

High deprived areas defined as being in the 4th or 5th quintile of the Townsend index of deprivation (2011). Metabolic syndrome was defined as the co-existence of medically diagnosed diabetes mellitus, high blood pressure, and obesity. Results are mean values, unless otherwise indicated, followed by 95% confidence interval.

Supplementary Table 2. Association between classification of amblyopia (i.e. i) all, ii) resolved, iii) persisting) and number of components relevant to the metabolic syndrome.

	Number of metabolic syndrome components		
	One vs None	Two vs None	Three vs None
	RRR (95% CI)	RRR (95% CI)	RRR (95% CI)
**All** confirmed amblyopia vs normal vision	1.22 (1.12; 1.34)	1.29 (1.13; 1.48)	1.40 (1.00; 1.95)
50-59 years vs 40-49 years	1.40 (1.30; 1.51)	2.18 (1.92; 2.48)	3.48 (2.38; 5.10)
60-75 years vs 40-49 years	1.87 (1.72; 2.04)	3.48 (3.03; 4.01)	6.62 (4.46; 9.82)
Male vs Female	1.25 (1.18; 1.34)	1.40 (1.26; 1.55)	1.88 (1.43; 2.48)
Other than white ethnic background vs white	1.42 (1.28; 1.58)	1.98 (1.68; 2.33)	1.82 (1.17; 2.82)
Townsend index vs 1st quintile (least deprived)			
2nd quintile	0.93 (0.84; 1.04)	1.31 (1.09; 1.58)	1.00 (0.59; 1.69)
3rd quintile	1.05 (0.95; 1.17)	1.36 (1.12; 1.64)	1.28 (0.77; 2.12)
4th quintile	1.12 (1.01; 1.24)	1.56 (1.30; 1.87)	1.56 (0.97; 2.51)
5th quintile (most deprived)	1.23 (1.11; 1.36)	2.02 (1.69; 2.41)	2.82 (1.79; 4.43)
**Resolved** amblyopia vs normal vision	1.26 (1.04; 1.53)	1.18 (0.87; 1.59)	1.10 (0.51; 2.38)
50-59 years vs 40-49 years	1.42 (1.31; 1.53)	2.15 (1.88; 2.46)	3.60 (2.39; 5.44)
60-75 years vs 40-49 years	1.90 (1.73; 2.08)	3.56 (3.07; 4.14)	8.07 (5.28; 12.34)
Male vs Female	1.25 (1.17; 1.34)	1.43 (1.28; 1.60)	1.97 (1.44; 2.68)
Other than white ethnic background vs white	1.43 (1.28; 1.60)	1.97 (1.66; 2.34)	2.05 (1.31; 3.21)

Townsend index vs 1st quintile (least deprived)			
2nd quintile	0.94 (0.84; 1.06)	1.35 (1.10; 1.66)	1.35 (0.74; 2.48)
3rd quintile	1.08 (0.96; 1.21)	1.38 (1.12; 1.69)	1.62 (0.90; 2.91)
4th quintile	1.15 (1.03; 1.28)	1.59 (1.30; 1.93)	1.81 (1.03; 3.19)
5th quintile (most deprived)	1.24 (1.11; 1.39)	2.02 (1.66; 2.47)	3.53 (2.06; 6.07)
**Persisting** amblyopia vs normal vision	1.22 (1.11; 1.34)	1.31 (1.14; 1.52)	1.45 (1.02; 2.05)
50-59 years vs 40-49 years	1.39 (1.29; 1.50)	2.19 (1.93; 2.50)	3.47 (2.36; 5.10)
60-75 years vs 40-49 years	1.86 (1.70; 2.02)	3.46 (3.00; 3.99)	6.77 (4.54; 10.1)
Male vs Female	1.26 (1.18; 1.34)	1.42 (1.27; 1.57)	1.80 (1.36; 2.37)
Other than white ethnic background vs white	1.42 (1.27; 1.58)	1.95 (1.65; 2.30)	1.87 (1.20; 2.91)
Townsend index vs 1st quintile (least deprived)			
2nd quintile	0.93 (0.83; 1.04)	1.29 (1.06; 1.56)	0.96 (0.57; 1.63)
3rd quintile	1.06 (0.95; 1.18)	1.35 (1.12; 1.64)	1.27 (0.77; 2.10)
4th quintile	1.12 (1.02; 1.25)	1.53 (1.28; 1.84)	1.48 (0.92; 2.39)
5th quintile (most deprived)	1.23 (1.10; 1.36)	2.04 (1.70; 2.44)	2.62 (1.66; 4.14)

$0.001 < p < 0.05$ (light shading)

$p < 0.001$ (dark shading)

Results are relative risk ratios (95% confidence intervals)) derived from multinomial logistic regression models, adjusted for all the covariates shown in table.

Supplementary Table 3. Baseline characteristics of the cohort for survival analysis.

		Amblyopia		
	Control (unaffected vision)	All	Resolved	Persisting
n (%)	17,860 (84.5)	3,269 (15.5)	605 (18.5)	2,664 (81.5)
Age, years	51.3 (51.2; 51.4)	57.1 (56.8; 57.3)	56.3 (55.7; 57.0)	57.3 (57.0; 57.5)
Male participants, n (%)	8,491 (47.5) (46.8; 47.5)	1,535 (47.0) (45.2; 48.7)	298 (49.3) (45.2; 53.3)	1,237 (46.4) (44.5; 48.4)
Other than white ethnic background, n (%)	1,985 (11.1) (10.7; 11.6)	100 (3.1) (2.5; 3.7)	16 (2.6) (1.6; 4.4)	84 (3.2) (2.5; 3.9)
Live in a highly deprived area, n (%)	7,179 (40.2) (39.5; 40.9)	1273 (38.9) (37.3; 40.6)	220 (36.4) (35.5; 40.4)	1053 (39.5) (37.7; 41.4)
Previous myocardial infarction, n (%)	207 (1.2) (1.0; 1.3)	63 (1.9) (1.5; 2.5)	9 (1.5) (0.7; 2.9)	54 (2.0) (1.5; 2.7)
Incident myocardial infarction, n (%)[1]	326 (1.8) (1.7; 2.1)	120 (3.7) (3.1; 4.5)	25 (4.2) (2.8; 6.2)	95 (3.6) (3.0; 4.5)
Previous stroke, n (%)	169 (0.9) (0.8; 1.1)	49 (1.5) (1.1; 2.0)	9 (1.5) (0.8; 2.9)	40 (1.5) (1.1; 2.1)
Incident stroke, n (%)[1]	182 (1.0) (0.9; 1.2)	64 (2.0) (1.5; 2.5)	6 (1.0) (0.4; 2.3)	58 (2.2) (1.7; 2.9)
Died during study period, n (%)	516 (2.9) (2.7; 3.1)	215 (6.6) (5.8; 7.5)	26 (4.3) (2.9; 6.3)	189 (7.1) (6.2; 8.2)

Highly deprived areas defined as being in the 4th or 5th quintile of the Townsend index of deprivation (2011). [1]Proportions for incident disease take, as denominator, those without a previous event.

Supplementary Table 4. Association between classification of amblyopia (i.e. i) all, ii) resolved, iii) persisting) and incident cardiovascular events and dementia.

	Myocardial infarction<br>HR (95% CI)	All-cause stroke<br>HR (95% CI)	All-cause death<br>HR (95% CI)
**All** confirmed amblyopia vs normal vision	1.38 (1.11; 1.72)	1.20 (0.89; 1.62)	1.36 (1.15; 1.60)
Per 10-year increase in age	2.08 (1.83; 2.36)	2.58 (2.16; 3.09)	2.80 (2.52; 3.11)
Male vs Female	3.03 2.45; 3.74)	1.20 (0.89; 1.62)	1.50 (1.29; 1.74)
Other than white ethnic background vs white	0.98 (0.67; 1.44)	1.31 (0.80; 2.14)	1.33 (1.00; 1.77)
Townsend index vs 1st quintile (least deprived)			
2nd quintile	0.92 (0.69; 1.23)	1.22 (0.83; 1.78)	1.15 (0.92; 1.45)
3rd quintile	0.81 (0.60; 1.09)	1.15 (0.78; 1.70)	1.11 (0.88; 1.41)
4th quintile	0.98 (0.73; 1.32)	0.95 (0.62; 1.45)	1.25 (0.98; 1.58)
5th quintile (most deprived)	1.35 (1.02; 1.79)	1.34 (0.89; 1.99)	1.65 (1.31; 2.07)

**Resolved** amblyopia vs normal vision	1.56 (1.03; 2.36)	0.62 (0.27; 1.40)	0.91 (0.61; 1.36)
Per 10-year increase in age	2.01 (1.75; 2.31)	2.62 (2.15; 3.19)	2.78 (2.47; 3.12)
Male vs Female	1.56 (1.03; 2.36)	1.62 (1.20; 2.17)	1.42 (1.20; 1.68)
Other than white ethnic background vs white	1.02 (0.69; 1.51)	1.33 (0.80; 2.21)	1.25 (0.92; 1.70)
Townsend index vs 1st quintile (least deprived)			
2nd quintile	0.93 (0.67; 1.30)	1.02 (0.66; 1.58)	1.08 (0.83; 1.41)
3rd quintile	0.85 (061; 1.20)	0.95 (0.61, 1.49)	1.05 (0.80; 1.37)
4th quintile	1.02 (0.73; 1.42)	0.94 (0.59; 1.49)	1.18 (0.90; 1.55)
5th quintile (most deprived)	1.32 (0.96; 1.83)	1.24 (0.79; 1.96)	1.53 (1.17; 1.99)
**Persisting** amblyopia vs normal vision	1.36 (1.07; 1.72)	1.33 (0.98; 1.81)	1.45 (1.21; 1.72)
Per 10-year increase in age	2.06 (1.81; 2.35)	2.58 (2.15; 3.09)	1.11 (1.10; 1.12)
Male vs Female	3.03 (2.44; 3.76)	1.51 (1.16; 1.95)	1.47 (1.26; 1.71)
Other than white ethnic background vs white	1.01 (0.69; 1.48)	1.31 (0.80; 2.15)	1.33 (1.00; 1.77)

Townsend index vs 1st quintile (least deprived)			
2nd quintile	0.94 (0.70; 1.26)	1.23 (0.84; 1.80)	1.14 (0.90; 1.44)
3rd quintile	0.80 (0.59; 1.10)	1.09 (0.73; 1.63)	1.06 (0.83; 1.35)
4th quintile	0.99 (0.73; 1.34)	0.93 (0.61; 1.44)	1.22 (0.96; 1.56)
5th quintile (most deprived)	1.29 (0.96; 1.72)	1.32 (0.88; 1.97)	1.60 (1.27; 2.02)

$0.001 < p < 0.05$

$p < 0.001$

Results are hazard ratios (95% confidence intervals)) derived from Cox proportional hazards models. The full model adjusted for all the covariates shown in table.

Supplementary Table 5. Balance statistics for the propensity matched analysis.

	Resolved								
	Standardized	differences	Variance	Standardized	differences	Variance	Standardized	differences	Variance
	Raw	Matched	Raw	Raw	Matched	Raw	Raw	Matched	Raw
**Diabetes**									
Age									
[50-59]yrs	0.010	1.17E-16	1.01	0.014	-2.33E-16	1.01	0.014	0.00E+00	1.01
[60-75]yrs	0.595	-2.23E-16	1.61	0.608	1.11E-16	1.61	0.595	1.12E-16	1.64
Sex									
Male	-0.001	-2.22E-16	1.00	-0.014	-1.11E-16	1.00	-0.011	-5.57E-16	1.00
Ethnicity									
Non-white	-0.319	1.22E-16	0.30	-0.316	8.04E-17	0.30	-0.320	-1.83E-16	0.30
Quintiles of deprivation									
2nd Q	0.030	-1.40E-16	1.05	0.026	-2.80E-16	1.04	0.032	2.09E-16	1.05
3rd Q	-0.019	7.14E-17	0.97	-0.013	-7.11E-17	0.98	-0.017	-2.14E-16	0.97
4th Q	-0.038	4.70E-16	0.95	-0.045	6.75E-17	0.94	-0.038	0.00E+00	0.95
5th Q (more ~)	-0.006	0	0.99	0.012	1.97E-16	1.02	-0.007	-2E-16	0.99
**High blood pressure**									
Age									
[50-59]yrs	0.014	0.00E+00	1.01	-0.003	-1.17E-16	1.00	0.018	-1.16E-16	1.01
[60-75]yrs	0.595	1.12E-16	1.64	0.537	0.00E+00	1.62	0.608	2.23E-16	1.64
Sex									
Male	-0.011	-5.57E-16	1.00	0.060	0.00E+00	1.01	-0.026	-2.23E-16	1.00
Ethnicity									
Non-white	-0.320	-1.83E-16	0.30	-0.336	0.00E+00	0.27	-0.317	-2.01E-16	0.30

Quintiles of deprivation									
2nd Q	0.032	2.09E-16	1.05	0.058	0.00E+00	1.09	0.026	7.01E-17	1.04
3rd Q	-0.017	-2.14E-16	0.97	-0.052	7.33E-17	0.92	-0.010	-7.08E-17	0.98
4th Q	-0.038	0.00E+00	0.95	0.001	-1.31E-16	1.00	-0.047	-2.03E-16	0.94
5th Q (more ~)	-0.007	-2E-16	0.99	-0.096	7.1E-17	0.87	0.011	-6.58E-17	1.01
**Obesity**									
Age									
[50-59]yrs	0.012	2.33E-16	1.01	-0.002	0.00E+00	1.00	0.015	1.17E-16	1.01
[60-75]yrs	0.594	-1.11E-16	1.61	0.530	1.12E-16	1.59	0.608	-1.11E-16	1.61
Sex									
Male	-0.001	-3.34E-16	1.00	0.063	0.00E+00	1.00	-0.015	-3.34E-16	1.00
Ethnicity									
Non-white	-0.320	0.00E+00	0.30	-0.332	0.00E+00	0.28	-0.317	-4.02E-17	0.30
Quintiles of deprivation									
2nd Q	0.030	-2.79E-16	1.05	0.049	0.00E+00	1.08	0.026	-2.80E-16	1.04
3rd Q	-0.018	2.86E-16	0.97	-0.042	-7.28E-17	0.94	-0.013	1.42E-16	0.98
4th Q	-0.034	5.36E-16	0.96	-0.004	1.31E-16	1.00	-0.041	3.37E-16	0.95
5th Q (more ~)	-0.008	-3.99E-16	0.99	-0.089	0	0.88	0.009	-2.63E-16	1.01
**Metabolic syndrome**									
Age									
[50-59]yrs	0.015	4.66E-16	1.01	-0.002	0	1.00	0.018	4.66E-16	1.01
[60-75]yrs	0.594	-4.46E-16	1.64	0.533	0.00E+00	1.62	0.607	-2.23E-16	1.64
Sex									
Male	-0.011	-3.34E-16	1.00	0.063	0	1.01	-0.027	-2.23E-16	1.00
Ethnicity									
Non-white	-0.314	1.01E-16	0.30	-0.330	0	0.27	-0.310	6.02E-17	0.31

Quintiles of deprivation									
2nd Q	0.030	-6.98E-17	1.05	0.053	0	1.09	0.025	-1.4E-16	1.04
3rd Q	-0.017	4.27E-16	0.97	-0.052	-7.32E-17	0.92	-0.010	4.25E-16	0.98
4th Q	-0.037	-6.71E-17	0.95	0.002	1.31E-16	1.00	-0.046	-6.75E-17	0.94
5th Q (more ~)	-0.007	-3.33E-16	0.99	-0.093	7.10E-17	0.88	0.011	-1.32E-16	1.02
**Vascular**									
Age									
[50-59]yrs	0.011	7.00E-16	1.01	-0.006	0.00E+00	1.00	0.014	2.33E-16	1.01
[60-75]yrs	0.594	-4.46E-16	1.61	0.533	1.12E-16	1.59	0.607	-1.11E-16	1.61
Sex									
Male	0.001	-8.89E-16	1.00	0.064	0.00E+00	1.00	-0.013	-6.68E-16	1.00
Ethnicity									
Non-white	-0.319	-2.03E-17	0.30	-0.330	0.00E+00	0.28	-0.317	-6.04E-17	0.30
Quintiles of deprivation									
2nd Q	0.031	-6.98E-17	1.05	0.055	-6.85E-17	1.09	0.026	-7.01E-17	1.04
3rd Q	-0.020	1.43E-16	0.97	-0.046	7.30E-17	0.93	-0.014	0.00E+00	0.98
4th Q	-0.037	0	0.95	-0.004	0	1.00	-0.044	-4.05E-16	0.94
5th Q (more ~)	-0.005	1.99E-16	0.99	-0.087	-7.04E-17	0.88	0.012	1.97E-16	1.02

Supplementary Table 6. Baseline characteristics of the cohort with retinal imaging data.

	**Control (n=35,061)**	**Amblyopia (n=623)**	**Fellow (n=663)**
Age, years	54.9 (54.8; 55.0)	55.7 (55.1; 56.3)	55.6 (55.0; 56.2)
Male participants, n (%)	15,650 (44.6) (44.1; 45.2)	262 (42.1) (38.1; 46.0)	271 (40.9) (37.1; 44.7)
Other than white ethnic background, n (%)	32,272 (92.0) (91.7; 92.3)	602 (96.6) (94.9; 97.9)	646 (97.4) (95.9; 98.5)
Live in a highly deprived area, n (%)	9,579 (27.3) (26.8; 27.8)	179 (28.7) (25.2; 32.5)	195 (29.4) (26.0; 33.0)
Smoking status, n (%)			
Never	20,067 (57.2) (56.7; 57.8)	347 (55.7) (51.7; 59.6)	372 (56.1) (52.2; 59.9)
Previous	11,662 (33.2) (32.8; 33.8)	214 (34.3) (30.6; 38.2)	216 (32.6) (29.0; 36.3)
Current	3,332 (9.5) (9.2; 9.8)	62 (10.0) (7.7; 12.8)	75 (11.3) (9.0; 14.0)
Alcohol drinking status, n (%)			
Never	1,522 (4.3) (4.1; 4.6)	22 (3.5) (2.2; 5.3)	22 (3.3) (2.1; 5.0)
Previous	1,137 (3.2) (3.1; 3.4)	21 (3.4) (2.1; 5.1)	21 (3.2) (2.0; 4.8)
Current	32,402 (92.4) (92.1; 92.7)	580 (93.1) (90.8; 95.0)	620 (93.5) (91.4; 95.3)
Body mass index, kg/m^2	27.1	27.3	27.3

	(27.0; 27.2)	(26.9; 27.7)	(26.9; 27.7)
Diabetes mellitus, n (%)	1,219 (3.5)	20 (3.2)	24 (3.6)
	(3.3; 3.7)	(2.0; 4.9)	(2.3; 5.3)
High blood pressure, n (%)	8,269 (23.6)	156 (25.0)	165 (24.9)
	(23.1; 24.0)	(21.7; 28.6)	(21.6; 28.4)
Refractive error, dioptres (D)	-0.46	1.68	0.83
	(-0.48; -0.44)	(1.37; 1.99)	(0.69; 1.07)
Visual acuity, logMAR	0.00	0.38	0.01
	(-0.002; 0.002)	(0.35; 0.41)	(-0.003; 0.02)
**Retinal layer thicknesses**			
mRNFL, µm	25.8	25.2	25.4 (2.4)
	(25.7; 25.8)	(24.8; 25.6)	(25.2; 25.6)
mGC-IPL, µm	90.1	87.9	89.2
	(90.0; 90.2)	(87.2; 88.6)	(88.2; 89.8)
**Retinovascular indices**			
Arteriolar caliber, µm	61.7	63.7	62.7
	(61.6; 61.8)	(63.2; 64.2)	(62.2; 63.2)
Venular caliber, µm	68	71.9	69.7
	(67.9; 68.1)	(61.6; 61.8)	(69.1; 70.3)
Fractal dimension, units	1.484	1.478	1.482
	(1.484; 1.484)	(1.475; 1.481)	(1.479; 1.485)
Distance tortuosity, units	3.43	3.61	3.43
	(3.42; 3.44)	(3.49; 3.73)	(3.32; 3.54)
**Optic nerve morphology**			
Cup height, units	62.9	61.8	62

	(62.7; 63.1)	(60.4; 63.2)	(60.7; 63.3)
Cup width, units	60.4	59.7	60
	(60.2; 60.6)	(61.6; 61.8)	(58.6; 61.4)
Disc height, units	132.2	130.5	131.3
	(132.0; 132.4)	(128.8; 132.2)	(129.6; 133.0)
Disc width, units	125.1	123.6	124.6
	(124.9; 125.3)	(121.8; 125.4)	(122.9; 126.3)

High deprived areas defined as being in the 4th or 5th quintile of the Townsend index of deprivation (2011). Data for optic nerve morphology were available for 32,525 controls, 544 cases and 583 fellow eyes. Results are mean values, unless otherwise indicated, followed by 95% confidence interval.

Supplementary Table 7. Association between amblyopia and retinal imaging outcome measures stratified into persisting (logMAR visual acuity ≥0.06) and resolved (logMAR visual acuity <0.06) amblyopia in the fully adjusted model.

	**Amblyopia**			
**Outcome**	**Persisting (*n* cases = 483)**		**Resolved (*n* cases = 140)**	
	**b (95% CI)**	***p*-value**	**b (95% CI)**	***p*-value**
**Retinovascular**				
Arteriolar caliber, per SD	0.00 (-0.09; 0.08)	0.92	0.17 (0.01; 0.32)	0.033
Venular caliber, per SD	0.24 (0.16; 0.33)	$2.7x10^{-8}$	0.37 (0.22; 0.53)	$3.1x10^{-6}$
Distance tortuosity, per SD	0.14 (0.05; 0.23)	0.002	0.04 (-0.12; 0.20)	0.63
Fractal dimension, per SD	-0.20 (-0.28; -0.12)	$2.7x10^{-6}$	-0.30 (-0.45; -0.15)	$1.3x10^{-4}$
**Retinal layer thickness**				
mRNFL, per µm	-0.11 (-0.36; 0.14)	0.37	-0.50 (-0.95; -0.05)	0.029
mGC-IPL, per µm	-2.97 (-3.68; -2.27)	$1x10^{-16}$	-1.70 (-2.99; -0.40)	0.010
**Optic nerve morphology**				
Cup height, per SD	-0.12 (-0.21; -0.02)	0.017	-0.07 (-0.25; 0.11)	0.43
Cup width, per SD	-0.09 (-0.19; 0.00)	0.05	0.09 (-0.09; 0.27)	0.30
Disc height, per SD	-0.17 (-0.27; -0.08)	$3.6x10^{-4}$	-0.14 (-0.32; 0.03)	0.11
Disc width, per SD	-0.17 (-0.27; -0.08)	$3.6x10^{-4}$	0.04 (-0.14; 0.22)	0.64

All models were adjusted for age, sex, ethnicity, socioeconomic deprivation, refractive error, diabetes mellitus, alcohol consumption, smoking history, and body mass index. Results are beta coefficients (95% confidence interval) derived from linear regression models. mGC-IPL: macular ganglion cell-inner plexiform layer, mRFNL: macular retinal nerve fiber layer, SD: standard deviation.
