# Supplementary material for: Associations between unilateral amblyopia in childhood and cardiometabolic disorders in adult life: a cross-sectional and longitudinal analysis of the UK Biobank
Source: eClinicalMedicine. 2024 Mar 7;70:102493. doi: 10.1016/j.eclinm.2024.102493 (PMC11056416; doi:10.1016/j.eclinm.2024.102493)
Supplement: UKBB Membership [file mmc2.docx]

UK Biobank & Eye Vision Consortium

| First names | Surnames |
| --- | --- |
| Naomi | ALLEN |
| Tariq | ASLAM |
| Denize | ATAN |
| Konstantinos | BALASKAS |
| Sarah | BARMAN |
| Jenny | BARRETT |
| Paul | BISHOP |
| Graeme | BLACK |
| Tasanee | BRAITHWAITE |
| Roxana | CARARE |
| Usha | CHAKRAVARTHY |
| Michelle | CHAN |
| Sharon | CHUA |
| Alexander | DAY |
| Parul | DESAI |
| Bal | DHILLON |
| Andrew | DICK |
| Alexander | DONEY |
| Cathy | EGAN |
| Sarah | ENNIS |
| Paul | FOSTER |
| Marcus | FRUTTIGER |
| John | GALLACHER |
| David (Ted) | GARWAY-HEATH |
| Jane | GIBSON |
| Jeremy | GUGGENHEIM |
| Chris | HAMMOND |
| Alison | HARDCASTLE |
| Simon | HARDING |
| Ruth | HOGG |
| Pirro | HYSI |
| Pearse | KEANE |
| Sir Peng Tee | KHAW |
| Anthony | KHAWAJA |
| Gerassimos | LASCARATOS |
| Thomas | LITTLEJOHNS |
| Andrew | LOTERY |
| Robert | LUBEN |
| Phil | LUTHERT |
| Tom | MACGILLIVRAY |
| Sarah | MACKIE |
| Savita | MADHUSUDHAN |
| Bernadette | MCGUINNESS |
| Gareth | MCKAY |
| Martin | MCKIBBIN |
| Tony | MOORE |
| James | MORGAN |
| Eoin | O'SULLIVAN |
| Richard | ORAM |
| Chris | OWEN |
| Praveen | PATEL |
| Euan | PATERSON |
| Tunde | PETO |
| Axel | PETZOLD |
| Nikolas | PONTIKOS |
| Jugnoo | RAHI |
| Alicja | RUDNICKA |
| Naveed | Sattar |
| Jay | SELF |
| Panagiotis | SERGOUNIOTIS |
| Sobha | SIVAPRASAD |
| David | STEEL |
| Irene | STRATTON |
| Nicholas | STROUTHIDIS |
| Cathie | SUDLOW |
| Zihan | SUN |
| Robyn | TAPP |
| Dhanes | THOMAS |
| Emanuele | TRUCCO |
| Adnan | TUFAIL |
| Ananth | VISWANATHAN |
| Veronique | VITART |
| Mike | WEEDON |
| Katie | WILLIAMS |
| Cathy | WILLIAMS |
| Jayne | WOODSIDE |
| Max | YATES |
| Yalin | ZHENG |
| Mervyn | THOMAS |
